# Editorial: The NAFLD-MAFLD conundrum

**DOI:** 10.3389/fendo.2023.1267341

**Published:** 2023-09-12

**Authors:** Andrea Dalbeni, Rahul Kumar, Somaya Albhaisi

**Affiliations:** ^1^ General Medicine C and Liver Unit, Medicine Department, Azienda Ospedaliera Universitaria Integrata Verona, Univerity of Verona, Verona, Italy; ^2^ Changi General Hospital Singapore and DUKE-NUS Academic Medical Center, Singapore, Singapore; ^3^ Department of Internal Medicine, Virginia Commonwealth University, Richmond, VA, United States

**Keywords:** MAFLD, MASLD, NAFLD (non-alcoholic fatty liver disease), liver, diabetes mellitus, BMI - body mass index, dyslipidemia

The aim of our Research Topic for Frontiers in Endocrinology-Gut Endocrinology, named the “*NAFLD-MAFLD Conundrum*”, is to discuss the current debate on renaming nonalcoholic fatty liver disease (NAFLD) to metabolic dysfunction-associated fatty liver disease (MAFLD) and the implications of changing terminology, summarizing the current state of knowledge regarding MAFLD and anticipating the necessity for a new nomenclature which is metabolic dysfunction-associated steatotic liver disease (MASLD) (1) given in the EASL summit in June 2023.

Overall, four original articles, a systematic review and two narrative reviews have been published in this Research Topic.

The importance of a new definition is well established in the systematic review by Wen et al. which included 10 cohort studies and showed that patients diagnosed with MAFLD alone had higher cardiovascular mortality than those diagnosed with NAFLD alone.

Our Research Topic aimed to identify the hot topics and new trends in the gut-liver axis in NAFLD/MAFLD research starting from the bibliometric analysis conducted by Liao et al., where they observed that the number of publications on MAFLD increased dramatically, especially in the last three years. In particular, gut microbiota became an important research direction for etiological and therapeutic investigations into MAFLD, as well as, insulin resistance, a key factor in studying the development of MAFLD. They concluded that liver fibrosis was an important focus of disease development.

Another bibliometric analysis by Yang et al. confirmed the previous analysis revealing that gut microbiota, inflammation, insulin resistance, short-chain fatty acids, and randomized controlled trials will be the new focus of research in the field.

Li et al. in a cross-sectional study among 4,195 participants aimed to evaluate the diagnostic efficacy of different anthropometric indices, including body mass index (BMI), waist circumference (WC), waist-to-height ratio (WHtR), waist-to-hip ratio (WHR), cardiometabolic index (CMI), triglyceride-glucose (TyG) index, hepatic steatosis index (HSI), lipid accumulation product (LAP), body roundness index (BRI), visceral fat index (VAI), abdominal volume index (AVI), cone index (CI), and body fat index (BAI), in adults with MAFLD to identify the best cut-off point for diagnosis of MAFLD in United States adults. In particular, the authors concluded that LAP may be a sensitive marker for diagnosing MAFLD in United States adults.

Another important parameter taken into account by Xiao et al. is the skeletal muscle mass (SMM). In the same cohort of Li et al., the authors demonstrated that the distribution of SMM differently affected MAFLD and significant fibrosis in the sex groups. Higher appendicular SMM was associated with a lower risk of MAFLD, while the risk of significant fibrosis in females was increased with the trunk SMM. Concluding the two published reviews focused the attention on molecular characterization of NAFLD/MAFLD (Che et al.) and on the latest treatments (Rong et al.).

We hope that this Research Topic will help to produce new evidence about NAFLD/MAFLD and contribute to better understand the new definition of MASLD, in order to clear the complexity of this disease.

## Author contributions

AD: Conceptualization, Writing – original draft. RK: Writing – review & editing. SA: Validation, Writing – review & editing.
